# Cadmium affects autophagy in the human intestinal cells Caco-2 through ROS-mediated ERK activation

**DOI:** 10.1007/s10565-021-09655-4

**Published:** 2021-09-28

**Authors:** Myriam Mireault, Yong Xiao, Benoît Barbeau, Catherine Jumarie

**Affiliations:** 1 Département des Sciences Biologiques, Groupe TOXEN, Université du Québec à Montréal, C.P. 8888, succ Centre ville, Montréal, Québec H3C 3P8 Canada; 2 Département des Sciences Biologiques, centre CERMO-FC, Université du Québec à Montréal, Montréal, Québec Canada

**Keywords:** Cadmium, Autophagy, ERK signaling, Reactive oxygen species, Intestinal cells

## Abstract

Cadmium is a toxic metal that enters the food chain. Following oral ingestion, the intestinal epithelium has the capacity to accumulate high levels of this metal. We have previously shown that Cd induces ERK1/2 activation in differentiated but not proliferative human enterocytic-like Caco-2 cells. As autophagy is a dynamic process that plays a critical role in intestinal mucosa, we aimed the present study 1) to investigate the role of p-ERK1/2 in constitutive autophagy in proliferative Caco-2 cells and 2) to investigate whether Cd-induced activation of ERK1/2 modifies autophagic activity in postconfluent Caco-2 cell monolayers. Western blot analyses of ERK1/2 and autophagic markers (LC3, SQSTM1), and cellular staining with acridine orange showed that ERK1/2 and autophagic activities both decreased with time in culture. GFP-LC3 fluorescence was also associated with proliferative cells and the presence of a constitutive ERK1/2-dependent autophagic flux was demonstrated in proliferative but not in postconfluent cells. In the latter condition, serum and glucose deprivation triggered autophagy via a transient phosphorylation of ERK1/2, whereas Cd-modified autophagy via a ROS-dependent sustained activation of ERK1/2. Basal autophagy flux in proliferative cells and Cd-induced increases in autophagic markers in postconfluent cells both involved p-ERK1/2. Whether Cd blocks autophagic flux in older cell cultures remains to be clarified but our data suggest dual effects. Our results prompt further studies investigating the consequences that Cd-induced ERK1/2 activation and the related effect on autophagy may have on the intestinal cells, which may accumulate and trap high levels of Cd under some nutritional conditions.

**Graphical abstract:**

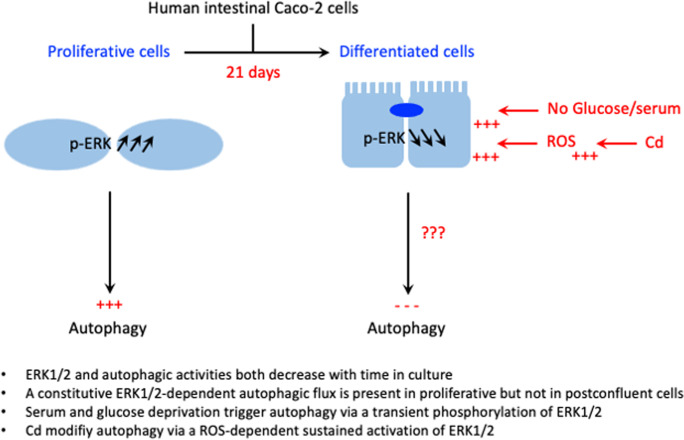

## Introduction

Cadmium is a widespread highly toxic metal classified as a type I carcinogen responsible for renal dysfunction, osteoporosis, immunosuppression and hepatotoxicity. Cadmium enters the food chain and, except for workers in mining industry and cigarette smokers, chronic oral absorption of contaminated food products represents the main exposure source for the general population (Andersen et al. 2004; Rani et al. 2014). Following ingestion, the gut epithelium represents the first protective barrier against Cd toxicity. Indeed, the oral bioavailability of Cd is less than 5% (WHO 1992). However, this results from the high capacity of intestinal cells to trap the accumulated metal from the lumen, with poor release into the bloodstream (Jumarie et al. 1999). According to our previous data, high levels of Cd were measured in gut tissue of animals fed with contaminated food at dosage close to the tolerable daily intake (Hiratsuka et al. 1999; Eklund et al. 2004). The intestinal epithelium therefore represents one of the first target tissue of the ingested Cd. Intestinal toxicity of Cd includes disruption of intercellular junctions, paracellular leakage and inflammation (for review see Tinkov et al. 2018). Cadmium may also modify gut immunity and microbiota, which could have clinical impact in the context of immune diseases such as inflammatory bowel diseases (Breton et al. 2016). Depending of the exposure condition, Cd may modify the cell cycle, and promote tumorigenicity or apoptosis (for review see Pius 2009). Indirectly, Cd also favors reactive oxygen species (ROS) formation, leading to oxidative stress, or minimally to a redox imbalance (Liu et al. 2009). Redox signaling is involved in the regulation of biochemical processes. The function of receptors, kinases, phosphatases, proteases, adhesion molecules, and transcription factors may be modulated via redox signaling (Forman et al. 2014). Thus, ROS are probably involved in many cellular response following exposure to Cd. Cadmium may also modify signaling cascades, including the extracellular signal-regulated kinases (ERK1/2), c-JUN N-terminal kinase (JNK) and p38 kinase in various cell types (Thévenod and Lee 2013).

Macroautophagy (so-called autophagy) is a process of autophagocytosis of cytosolic components and damaged organelles, and is essential to tissue homeostasis (Levine and Klionsky 2004). Autophagy is induced by starvation, metabolic stress, pathogenic and genotoxic conditions. It involves specific factors including light chain 3 (LC3) and sequestosome 1 (SQSTM1/p62) (Mehrpour et al. 2010). Once synthesized LC3, the mammalian homologue of yeast Atg8, is processed by cleavage and yields the cytosolic form LC3-I. Upon autophagy activation, LC3-I is subjected to lipidation with phosphatidylethanolamine (PE) to form LC3-II, which is then inserted in phagophores) and involved in autophagosome elongation. Although heavier, the PE-conjugated form of Atg8/LC3 (14–16 kDa) has faster electrophoretic mobility in SDS-PAGE compared to the nonlipidated form LC3-I (16–18 kDa). SQSTM1 (62 kDa) is involved in the degradation process of ubiquitinylated proteins. It binds to these proteins and forms aggregates that are incorporated into autophagosome by direct association with LC3-II. Since SQSTM1 and LC3 are located in the membrane of the autophagosome, they are ultimately subjected to degradation by autophagy. However, upon autophagy activation, levels of SQSTM1 generally decrease while increases in LC3-II are observed as a result of LC3-I conversion to LC3-II (Klionsky et al. 2016). In the gut epithelium, autophagy is associated with proliferative cells (which express high levels of active phosphorylated ERK1/2) in the lower third of the crypts (Groulx et al. 2012).

In previous studies, we have shown that a 24 h exposure to subcytotoxic levels of Cd activated ERK1/2 and p38 kinase in enterocytic-like Caco-2 cells (Mantha and Jumarie 2010; Gebraël and Jumarie 2015; Lemaire et al. 2020). This phenomenon was observed over a small range of metal concentrations and was characterized by a hormesis-like response optimal at 10 µM Cd. Moreover, this effect was cell state specific, being observed in the differentiated cells exclusively; Cd did not stimulate ERK1/2 in undifferentiated cells. Interestingly, as it occurs along the crypt-villus axis in the intestinal tissue, in Caco-2 cells, active p-ERK1/2 is mainly expressed in undifferentiated cultures, while its activity decreases when Caco-2 cells differentiate upon confluency (Aliaga et al. 1999; Gauthier et al. 2001). The growth-related differentiation of Caco-2 cells has been well characterized, and this cell line, which undergoes spontaneous enterocytic differentiation after confluency, has been widely used to study intestinal functions, drug permeability and xenobiotic biotransformation (Jumarie and Malo 1991; Vachon and Beaulieu 1992; Sun and Pang 2008; Artursson et al. 2012). A number of studies reported that ERK1/2 signaling may modulate autophagy activity but with different resulting effects since p-ERK1/2 can promote autophagy (Wang et al. 2009b; Meng et al. 2015; Bastola et al. 2017) or, on the contrary, lead to its inhibition (Kinsey et al. 2019; Vukelic et al. 2020; Qiao et al. 2021). As such, the role played by ERK1/2 signaling in autophagy still deserves to be investigated, especially in cell types that undergo differentiation with variation in the level of ERK1/2 ativation. It has also been shown that ROS, alone, or resulting from exposure to Cd, may modify autophagy activity (Wang et al. 2009a, 2015; Wong et al. 2010; Li et al. 2015; Bastola et al. 2017). Our previous studies also provided evidence for the involvement of ROS in Cd-induced ERK1/2 activation (Lemaire et al. 2020). To our knowledge, the link between ERK1/2 activation in cells with constitutive low levels of p-ERK1/2, ROS and autophagy, along with the duration of exposure to Cd has been poorly investigated. The objectives of the present study were 1) to investigate the role of p-ERK1/2 in constitutive autophagy in proliferative Caco-2 cells and 2) to investigate whether Cd-induced activation of ERK1/2 may modify autophagic activity in postconfluent Caco-2 cell monolayers.

## Materials and methods

### Cell culture

The human enterocytic-like cells Caco-2 were used between passages 201 and 220 (Grasset et al. 1984). Cells were maintained in 75 cm^2^ flasks (Corning Inc., Corning, NY, USA) at 37 °C in a 5% CO_2_ humidified atmosphere in Dulbecco’s modified eagle essential minimum medium (DMEM) (Gibco, ThermoFisher Scientific, Life Technologies, Waltham, MA, USA) supplemented with 15% inactivated fetal bovine serum (FBS) (Wisent In., St-Bruno, QC, Canada), 0.1 mM non-essential amino acids, and penicillin–streptomycin (50 U/ml to 50 µg/ml) (Gibco Life Technologies), pH 7.3. Cultures were passaged by trypsinization (0.05% trypsin-0.53 mM EDTA) every week and were seeded at a density of 1.2 × 10^4^ cells/cm^2^ in 60 × 20 mm or 100 × 20 mm diameter culture dishes (Sarsted, Nümbrecht, Germany), 96-well or 24-well plates (Sarstedt), or 15 µ-slide 4-well coverslip (Ibidi USA, ThermoFisher Scientific) for subsequent experiments. The culture medium was changed every 2 days and the cells were maintained for 21 days to allow functional differentiation (Jumarie and Malo 1991; Vachon and Beaulieu 1992). Caco-2 cells undergo an exponential growth phase until the culture dish confluence is reached, i.e. around day 7 of culture at the above mentioned seeding density. Then, proliferation stops and the cell monolayer begins to differentiate (Jumarie and Malo). In the present study, 7-day-old cells refer to (almost) confluent but undifferentiated cell monolayers, whereas 21-day-old cells are postconfluent differentiated cell monolayers.

### Immunoblotting

Seven and 21-day-old cell cultures were rinsed twice with FBS-free DMEM before incubation with 10 µM Cd (added as CdCl_2_, MilliporeSigma 99.99% purity) for ERK1/2 activation, as shown in our previous studies (Mantha and Jumarie 2010; Gebraël and Jumarie 2015; Lemaire et al. 2020). Cells were next incubated with or without 20 µM of the MEK1/2 (upstream ERK1/2) inhibitor U0126 (Calbiochem®, MilliporeSigma Burlington MA, USA), 5 mM 3-methyladenine (3-MA), or 50 nM bafilomycin A1 (MilliporeSigma), two autophagy inhibitors, in FBS-free medium for 24 h at 37ºC. For some experiments, cells were also exposed to 1 mM N-acetylcysteine (NAC, MilliporeSigma) (antioxidant), or 3 mM L-buthionine-sulfoximine (BSO, MilliporeSigma), which inhibits glutathione synthesis (pro-oxidant). In some experiments, cells were glucose-starved 24 h or 48 h in FBS-free medium.

Cells were then harvested in an ice-cold hypotonic lysis buffer, passed through a 26-G needle and centrifuged at 10 000 g for 25 min at 4ºC. The pellet was discarded and 20 μg of resulting cellular extract were mixed with 62.5 mM Tris–HCl buffer (2% SDS, 10% glycerol, 5% β-mercaptoethanol, pH 6.8), heated to 95ºC for 5 min, and separated by electrophoresis in 10% (or 12.5%, pH 9.1, in some experiments related to LC3-I and LC3-II) sodium dodecyl sulphate–polyacrylamide gel (SDS–PAGE). Proteins were electrotransfered to PVDF membranes (Millipore, Billerica, MA), which were blocked by a 1-h incubation with Tris-buffered saline (TBS) containing 0.1% Tween-20 and 5% non-fat milk at room temperature. Membranes were incubated overnight at 4ºC with the primary antibodies (Cell Signaling Technology, Inc. Danvers, MA) specific to p44/42MAPkinase (1:1000) (9102), phospho-p44/42MAPkinase (Thr202/Tyr204) (1:1000) (9101), LC3B (1:500) (2775), β-actin (1:1000) (4967) and (Abcam Inc. Toronto, ON Canada) SQSTM1/p62 (1:2000) (ab56416). Membranes were then incubated with a mixture of secondary antibodies (Cell Signaling Technology): horseradish peroxidase (HRP)-conjugated goat anti-rabbit IgG (1:2000) (7074) or goat anti-mouse IgG (1:1000) (7074) and HRP-conjugated anti-biotin (1:1000) (7075) antibodies for 1 h. In some experiments, anti-GAPDH (6C5) (1:1000) (SC-32233) (Santa Cruz Biotech) and sheep anti-mouse IgG (1:1000) (NA931) (Amersham GE Healthcare) antibodies were used for loading control. Membranes were revealed with HyGLO (Denville Scientific, Metuchen, NJ, USA) and visualized using a Chemiluminescence-Fluorescence and Advanced Fluorescence Fusion FX7 instrument (Montreal Biotech Inc. Laboratory Equipment). Densitometry obtained with the treated cell samples was normalized relative to that of β-actin or GAPDH and compared to control cells using ImageJ 1.48p software (Wayne Rasband National Institutes of Health, USA).

### Immunostaining fluorescence

Twenty-day-old cell cultures were rinsed twice with FBS-free DMEM before incubation in the presence of 10 µM Cd with or without 20 µM U0126, 50 nM bafilomycin A1 or 1 mM NAC in FBS-free medium for 24 h at 37ºC. Thereafter, cells were fixed in ice-cold 4% paraformaldehyde for 10 min at room temperature on a slow rotatory shaker. Cells were next washed four times with PBS–0.1% Triton X-100. The cells were incubated in blocking solution (3% BSA, 5% milk, 50% FBS, 0.1% Triton X-100, and 0.05% NaN_3_ in PBS) for 2 h, washed and incubated in the presence of anti-LC3 antibodies in PBS solution (containing 3% BSA, 0.1% Triton X-100, and 0.05% NaN_3_) overnight at 4 °C. Cells were next washed with cold PBS–0.1% Triton X-100 and incubated with goat anti-rabbit IgG coupled to Alexa Fluor 488 (1:1000) (λ_ex_: 488 nm, λ_em_: 525/50 nm, Life technologies) in the PBS solution described above for 1 h at room in dark. Cells were rinsed with PBS four times, incubated with 0.3 µM DAPI (λ_ex_: 405 nm, λ_em_: 450/50 nm) for 5 min, rinsed again and maintained in PBS for visualization with a Nikon A1R confocal laser microscope system (Japan 2011) using a Plan Apo 60 × oil objective.

### GFP-LC3 transient cells transfection

Seven-day-old cell cultures were transfected with 500 ng GFP-LC3 expression vector using lipofectamine™ 3000 reagent (ThermoFisher Scientific) in Opti-MEM™-reduced serum medium for 15 min at room temperature according to manufacturer’s instructions. Cells were then washed with PBS, and GFP fluorescence (λ_ex_: 488 nm, λ_em_: 525/50 nm) was visualized with a Nikon A1R confocal laser microscope system (Japan 2011) using a Plan Apo 10x/0.45 DIC L or a Plan fluor 20x/0.75 Mlmm DIC N2 objective.

### Acid autolysosome visualization

Seven and 21-day-old cell cultures were rinsed twice with FBS-free DMEM before incubation in the presence of 10 µM Cd with or without 20 µM U0126, 5 mM 3-MA, 50 nM bafilomycin A1, 1 mM NAC, or 3 mM BSO in FBS-free medium for 24 h at 37ºC. In some experiments, cells were glucose-starved 24 h or 48 h in FBS-free medium. Thereafter, cells were rinsed with PBS and incubated in PBS with 15 µM acridine orange (AO) (MilliporeSigma) for 15 min at 37ºC in the dark. After rinsing with PBS, fluorescence (λ_ex_: 488 nm, λ_em_: 525 nm for green DNA staining, λ_em_: 595 nm for red acid vacuoles staining) was visualized with a Nikon A1R confocal laser microscope system (Japan 2011) using a Plan Apo 10x/0.45 DIC L objective.

### Statistical and data analyses

Data are shown as means ± SD obtained from 3 to 8 independent cell cultures. Of note, LC3-II/LC3-I ratios are means estimated from several cell cultures (not ratios of mean values for individual LC3-II and LC3-I). Also levels of LC3-II and LC3-I were normalized to that of β-actin or GAPDH in estimation of LC3-II/LC3-I ratio. Depending on the number of experiments for each condition, the requirement of normality was not always respected. Hence, comparisons between experimental conditions were analyzed using nonparametric Wilcoxon-Mann–Whitney tests. Statistical analyses were performed using JMP Pro 14.0.0 (SAS Institute Inc., Cary, California, USA). Statistical significance was assessed at *p* ≤ 0.1.

## Results

### Autophagic and ERK activities as a function of days in culture

**Fig. 1:**
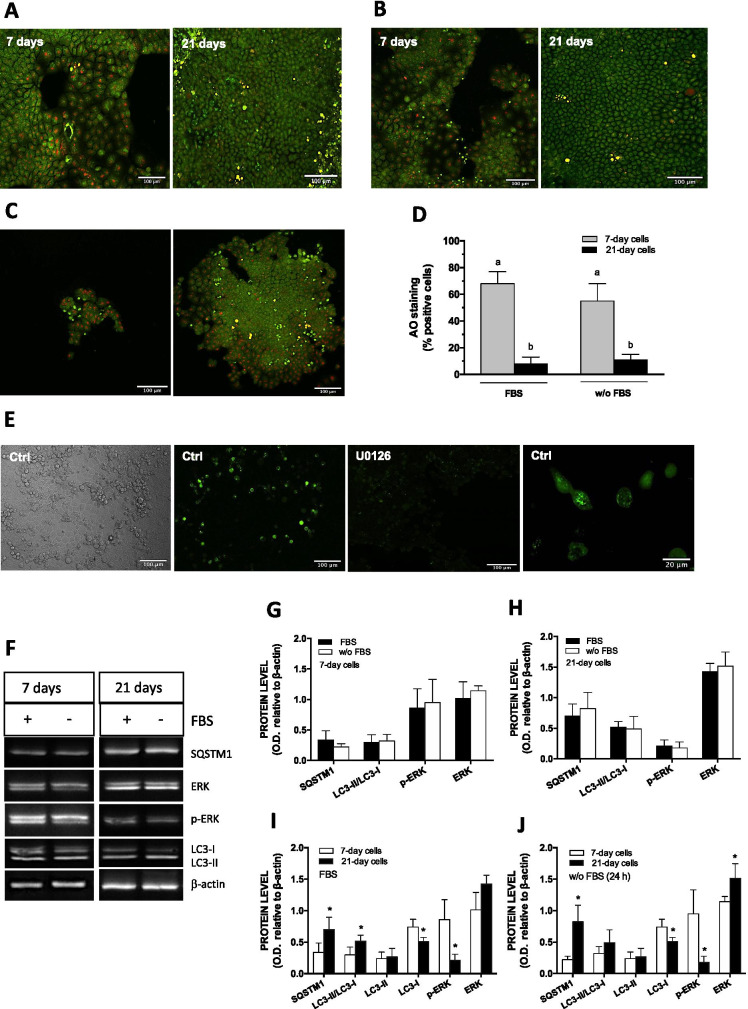
Autophagy as a function of time of culture. AO staining in 7-day and 21-day-old cells without (**A**) or following a 24 h FBS deprivation (**B**). (**C**) Examples of cell monolayer with proliferative red AO staining at the periphery. (**D)** Quantification of AO-positive cells. (**E)** LC3 punctae in cells grown in the presence of FBS for 6 days, transfected with 500 ng GFP-LC3 plasmid DNA for 15 min and then treated or not with 20 µM U0126 in FBS-free medium for 24 h before visualization. (**F–J**) Western blot and quantification of ERK1/2 and autophagy related proteins in 7-day and 21-day-old cells following or not a 24 h FBS deprivation. Scale bar: 100 µm (except last picture in E: 20 µm). Data are means ± SD estimated from 3 to 8 independent cell cultures. *Significantly different (*P* ≤ 0.1) compared to levels estimated in 7-day-old cells. Different letters indicate significant differences (*P* ≤ 0.1)

In previous studies, we have shown that a 24 h exposure to 10 µM Cd activated ERK1/2 in 21-day-old but not 7-day-old cell cultures (Mantha and Jumarie 2010; Gebraël and Jumarie 2015). As Cd complexation to albumin may lower the level of cellular uptake, incubations were conducted in the absence of FBS (Pham et al. 2004). Thus, autophagy and ERK1/2 activities were first studied as a function of days in culture in presence or absence of FBS. As a first step, we used acridine orange (AO) to address the extent of ongoing autophagy through detection of autolysosomes. The percentage of AO-positive cells decreased from 68% in 7-day-old cells to 8% in 21-day-old cells (Fig. 1A, D). Similar observations were obtained following a 24 h incubation in FBS-free medium before staining with AO: percentage of AO-positive cells lowered from 55 to 11% (Fig. 1B, D). Of note, in 7-day cultures, red puncta were more numerous in area with a high level of cellular proliferation whereas staining was much less present in dense area where cells stopped proliferating. This correlation was also demonstrated in regions with cell aggregates: central regions were devoid of red AO fluorescence, while at the periphery, where proliferation takes place, red staining was evident (Fig. 1C). Because AO red fluorescence is not exclusive to autophagolysosomes, additional experiments were conducted with 7-day cell cultures transfected with GFP-LC3 plasmid. The pattern of GFP fluorescence agreed with that of AO, and was associated with round cells surrounding mosaic monolayer regions characteristic of cells with tight junctions (Vachon and Beaulieu 1992) (Fig. 1E). These observations are in accordance with a higher autophagic activity associated with proliferative cells (Groulx et al. 2012). They also show that, in addition to autophagy markers, AO may be used to monitor autophagy in Caco-2 cells as a function of days in culture.

The effect of FBS depletion on protein levels of ERK1/2 as well as SQSTM1 and LC3, two markers of autophagic activity, was also analyzed. In 7-day-old and 21-day-old cells, a 24 h FBS starvation did not significantly modify the abundance of these proteins (Fig. 1F, G, H). For all culture condition, the levels of p-ERK1/2 were 3 to fourfold lower in 21-day cells compared to day 7. (Fig. 1F, I, J). Older cells expressed higher levels of SQSTM1 with higher LC3-II/LC3-I ratio, which were related to decreases in the level of LC3-I (no changes in LC3-II levels). Similar variations were observed in cells starved from FBS for 24 h although LC3-II/LC3-I ratio were not significantly different (Fig. 1F, J). These observations are discussed later. At the present step, our results showing higher level of SQSTM1 in older culture with the pattern of AO and GFP-LC3 fluorescence associated with proliferative cells exclusively suggest higher autophagic activity in undifferentiated cells with high levels of active p-ERK1/2. These variations were not modified by a 24 h FBS deprivation.

### ERK and autophagic flux in preconfluent Caco-2 cells

**Fig. 2:**
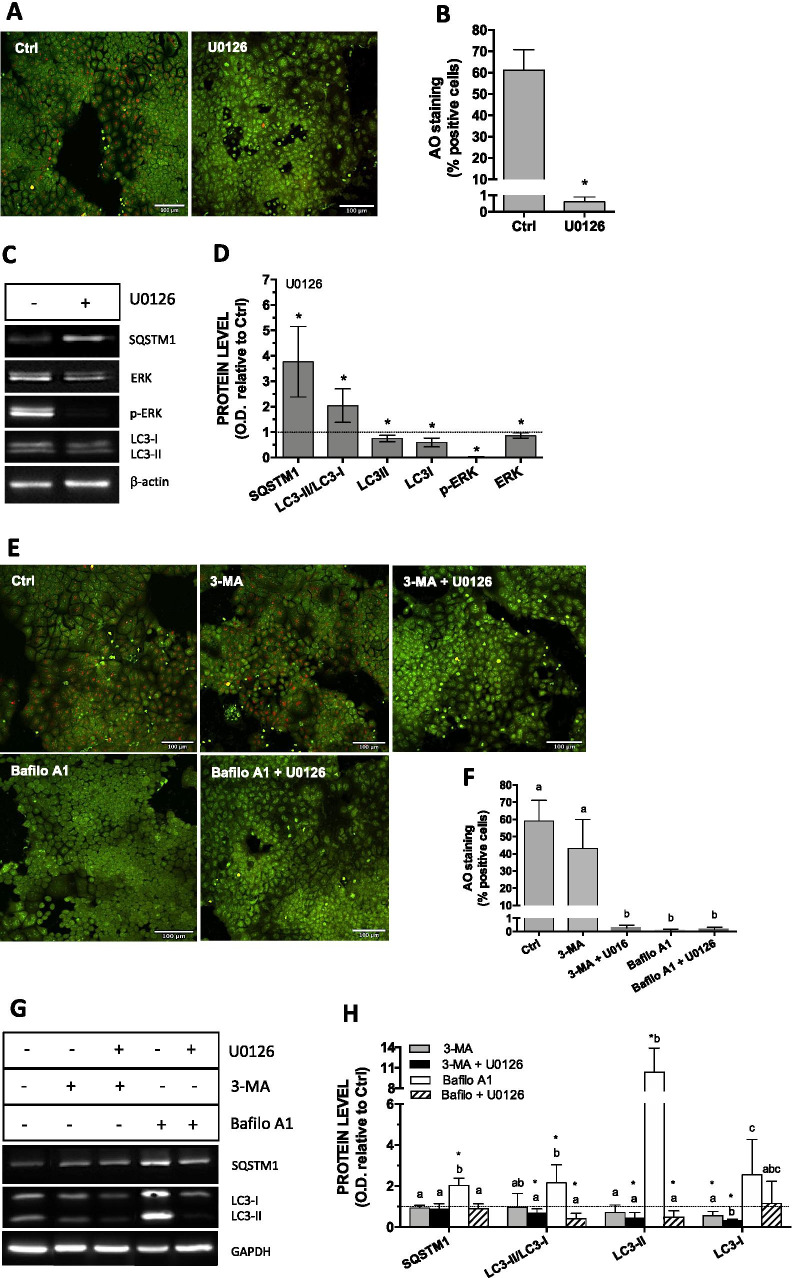
Involvement of p-ERK1/2 in the basal autophagic flux in 7-day-old cells following a 24 h FBS deprivation. (**A, E**) Visualization of acidic autolysosomes with the dye AO in the presence of inhibitors. (**B, F**) Quantification of AO-positive cells. (**C, D, G, H**) Representative Western blots and quantification of ERK1/2 and autophagy related proteins in 7-day-old cells following a 24 h FBS deprivation in the presence of 20 µM U0126, 5 mM 3-MA or 5 nM bafilomycin A1 relative to that of control cells (FBS-free medium alone for 24 h). Scale bar: 100 µm. Data are means ± SD estimated from 3 to 5 independent cell cultures. *Significantly different (*P* ≤ 0.1) compared to levels estimated in control cells. Different letters indicate significant differences (for the same protein in H) (*P* ≤ 0.1)

The hypothetical role of ERK in basal autophagic activity in 7-day-old cells was investigated using U0126, an inhibitor of MEK, which lies upstream to ERK. The percentage of AO-positive cells decreased from 60 to less than 1% in the presence of U0126 (Fig. 2A, B). Also, cells treatment with 20 µM U0126 lowered p-ERK1/2 level, while leading to higher levels of SQSTM1 probably resulting from lower degradation level (Fig. 2C, D). Both LC3-II and LC3-I decreased in the presence of U0126, but the reduction in LC3-I was more important than that of LC3-II (50% vs. 25%), resulting in a twofold increase in LC3-II/LC3-I ratio.

The autophagic flux along with ERK activity was also investigated using 3-MA which inhibits PI3K involved in the initiation of autophagy and prevents phagophore formation, and bafilomycin A1, which prevents autolysosome formation. 3-Methyladenine alone did not significantly modify the percentage of AO-positive cells, but the addition of U0126 lowered it from 60% to less than 1% (Fig. 2E, F). As expected, no AO red fluorescence was observed in the presence of bafilomycin A1, which inhibits H^+^-ATPase activity. Immunoblotting analyzes revealed no effect of 3-MA on the levels of SQSTM1, whether ERK/1/2 was inhibited or not (Fig. 2G, H). However, a twofold increase, reversed by U0126, was observed in the level of SQSTM1 in the presence of bafilomycin A1, which is in accordance with the fact that bafilomycin A1 inhibits autophagy flux. Similarly, although 3-MA did not modify the LC3-II/LC3-I ratio, bafilomycin A1 led to an increase of the ratio by twofold (Fig. 2G, H). Importantly, LC3-II increased tenfold when autolysosome formation (and thus protein degradation) was prevented by bafilomycin A1. However, this phenomenon was completely inhibited by U0126. In this case, the LC3-II/LC3-I ratio was even lower than control values. Altogether, these results reveal the presence of basal (constitutive) autophagic flux in undifferentiated Caco-2 cells being regulated by ERK1/2 signaling.

### Kinetic of Cd effect on autophagic markers in postconfluent Caco-2 cell monolayers

**Fig. 3:**
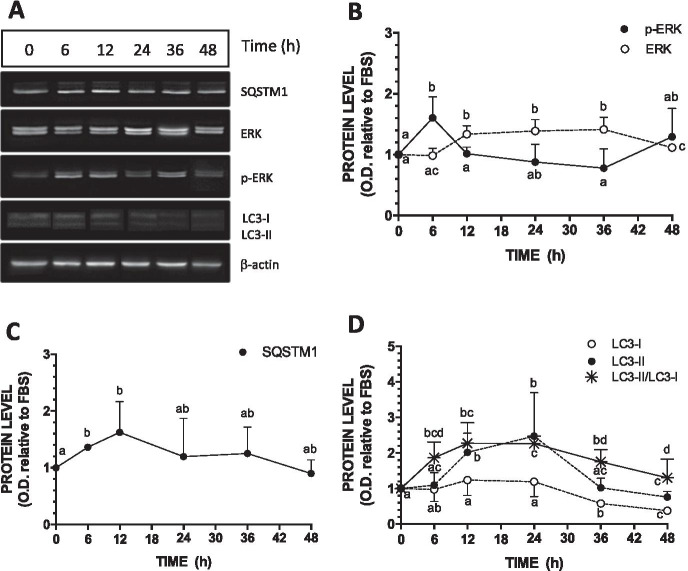
Effect of FBS deprivation on the levels of ERK1/2 and autophagic markers (LC3, SQSTM1) in 21-day-old cells. Cells were grown in the presence of FBS for 21 days and were then FBS-starved for the indicated period. **A.** Representative blot. **B–D.** Quantification of ERK1/2 and autophagy related proteins as a function of time in FBS-free medium. Data are means ± SD estimated from 3 to 4 independent cell cultures. For the same protein, different letters indicate significant differences (*P* ≤ 0.1)

**Fig. 4:**
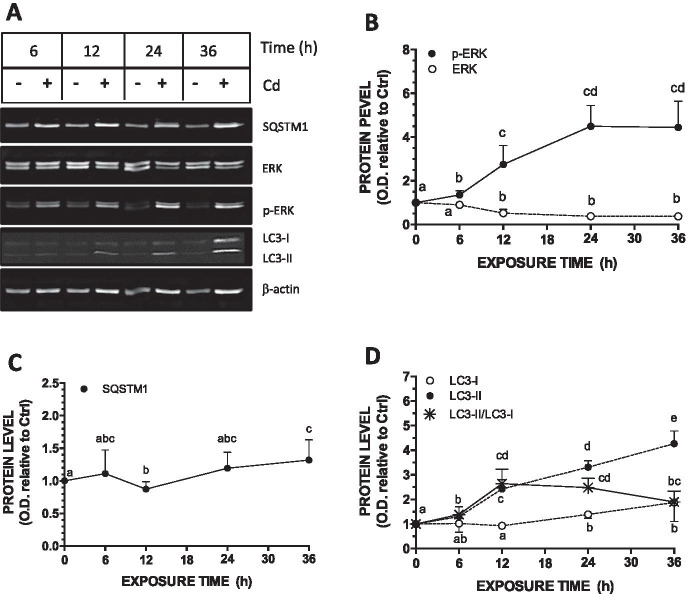
Effect of time of exposure to Cd on the levels of ERK1/2 and autophagic markers (LC3, SQSTM1) in 21-day-old cells. Cells were grown in the presence of FBS for 21 days and were then exposed to 10 µM Cd in FBS-free medium for the indicated period. **A.** Representative blot. **B-D.** Quantification of ERK1/2 and autophagy-related proteins as a function of time of exposure to Cd. Data are means ± SD estimated from 3 to 4 independent cell cultures. For the same protein, different letters indicate significant differences (*P* ≤ 0.1)

In previous studies, we have shown ERK1/2 activation in 21-day-old Caco-2 cells (in which p-ERK1/2 is normally very low) following a 24 h exposure to Cd (Mantha and Jumarie 2010; Gebraël and Jumarie 2015; Lemaire et al. 2020). To study the possible effect of Cd on autophagic activity in differentiated cells, we first confirmed that 24 h was the optimal exposure time. Because cells are exposed to Cd in the absence of FBS, kinetic studies on the effect of FBS deprivation were first conducted. A small but significant peak was observed in the level of p-ERK1/2 following 6 h of FBS deprivation, while levels became similar to those measured in the presence of FBS at 12 h and later time point (Fig. 3A, B). Contrary to p-ERK1/2, levels of total ERK1/2 increased slightly and plateaued at 12 h following FBS deprivation. The highest levels of SQSTM1 and LC3-II were observed at 6 and 12 h after FBS deprivation, respectively (Fig. 3A, C, D). The levels of LC3-I did not vary significantly during the first 24 h of incubation in the absence of FBS, but it decreased thereafter, resulting in a twofold increase in the LC3-II/LC3-I ratio observed up to 48 h. These results show that FBS deprivation may modulate autophagic activity. Therefore, in subsequent experiments on Cd, FBS-starved cells were used as control cells. Kinetic studies were then conducted with cells exposed to Cd. The maximal activation of ERK1/2 (fourfold increase in the levels of p-ERK1/2) was reached following a 24-h exposure to 10 µM Cd (Fig. 4A, B). Similar results were obtained with the levels of SQSTM1 although variations were more modest (Fig. 4A, C). The levels of LC3-I and LC3-II constantly increased during the first 24 h and 36 h of exposure to Cd (2- and fourfold, respectively), whereas the LC3-II/LC3-I ratio reached a peak at 12 h (Fig. 4A, D). A 24 h exposure to Cd was thus selected for subsequent experiments.

### Effect of Cd and glucose deprivation on autophagic activity in post-confluent Caco-2 cells

**Fig. 5:**
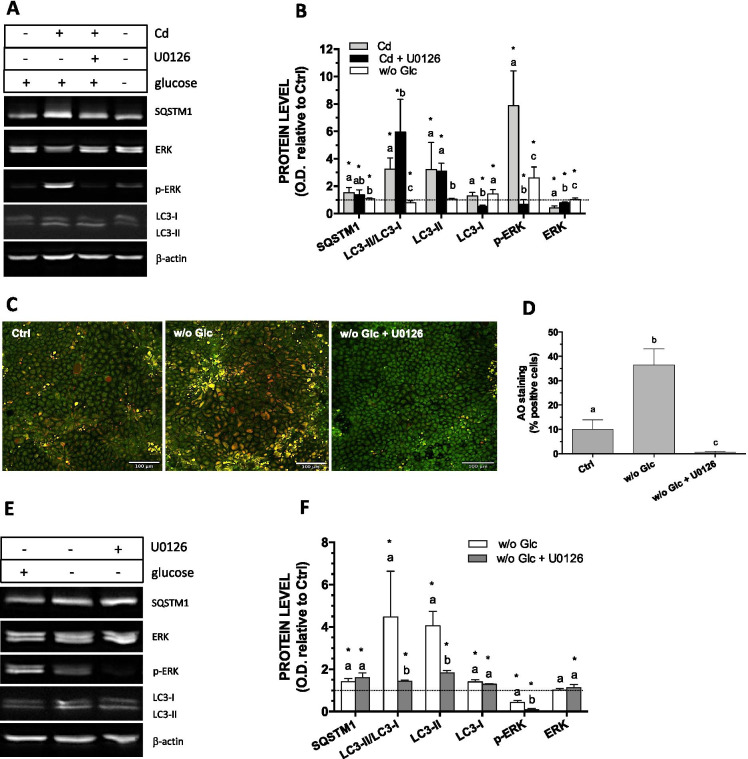
Comparison between the effect of Cd and glucose deprivation on autophagy markers in 21-day-old cells. (**A, B**) Representative Western blot and quantification of ERK1/2 and autophagy related proteins in 21-day-old cells glucose-starved or exposed to 10 µM Cd for 24 h relative to that of control cells (FBS-free medium alone for 24 h). (**C**) Visualization of acidic autolysosomes with the dye AO in cells glucose straved for 48 h. (**D**) Quantification of AO-positive cells following a 48-h glucose starvation. (**E, F**) representative Western blot and quantification of ERK1/2 and autophagy related proteins in 21-day-old cells glucose-starved for 48 h relative to that of control cells (FBS-free medium alone for 48 h). Scale bar: 100 µm. Data are means ± SD estimated from 3 to 8 independent cell cultures. *Significantly different (*P* ≤ 0.1) compared to levels estimated in control cells. Different letters indicate significant differences (for the same protein in B and F) (*P* ≤ 0.1)

The effect of Cd was first compared to that of glucose deprivation, which activates autophagy by inducing a nutritional stress, and which is believed to involve ERK1/2 activation (Roberts et al. 2014). A 24 h glucose starvation and a 24 h exposure to Cd both increased p-ERK1/2 levels (3- and eightfold, respectively) but only Cd succeeded in increasing the LC3-II/LC3-I ratio (Fig. 5). In fact, LC3-I remained constant whereas LC3-II increased suggesting a higher rate of LC3-I conversion to LC3-II (Fig. 5B). In contrast, a 24 h glucose deprivation increased LC3-I without modifying levels of LC3-II and lead to a lower LC3-II/LC3-I ratio. The Cd-induced ERK1/2 activation was completely inhibited by the presence of U0126, while the increase in LC3-II/LC3-I ratio was doubled in the presence of the ERK1/2 inhibitor, as a result of lower level of LC3-I. To get a better appreciation of the effect of glucose deprivation on autophagy, duration of starvation was increased to 48 h. This cell treatment led to a 3.5-fold increase in the percentage of AO-positive (Fig. 5C, D) and to a near fivefold higher LC3-II/LC3-I ratio which was clearly related to higher LC3-I conversion to LC3-II (Fig. 5F). The fluorescence and the increase in LC3-II were both inhibited by U0126. Interestingly, contrary to what was observed following glucose starvation of 24 h, no increase in p-ERK1/2 levels were observed at 48 h of starvation. ERK1/2 activation was even lower compared to control conditions. This could reflect the existence of a sequence in the events leading to autophagy, and suggest that the activation of ERK1/2 in inducing autophagy would be prerequisite but transient. A 48 h glucose deprivation also slightly increased SQSTM1 as did a 24 h exposure to Cd. The data suggest that Cd and glucose deprivation distinctly modulate autophagy in post-confluent Caco-2 cells through ERK1/2 activation.

### Effect of Cd on autophagic flux in post-confluent Caco-2 cell monolayers

**Fig. 6:**
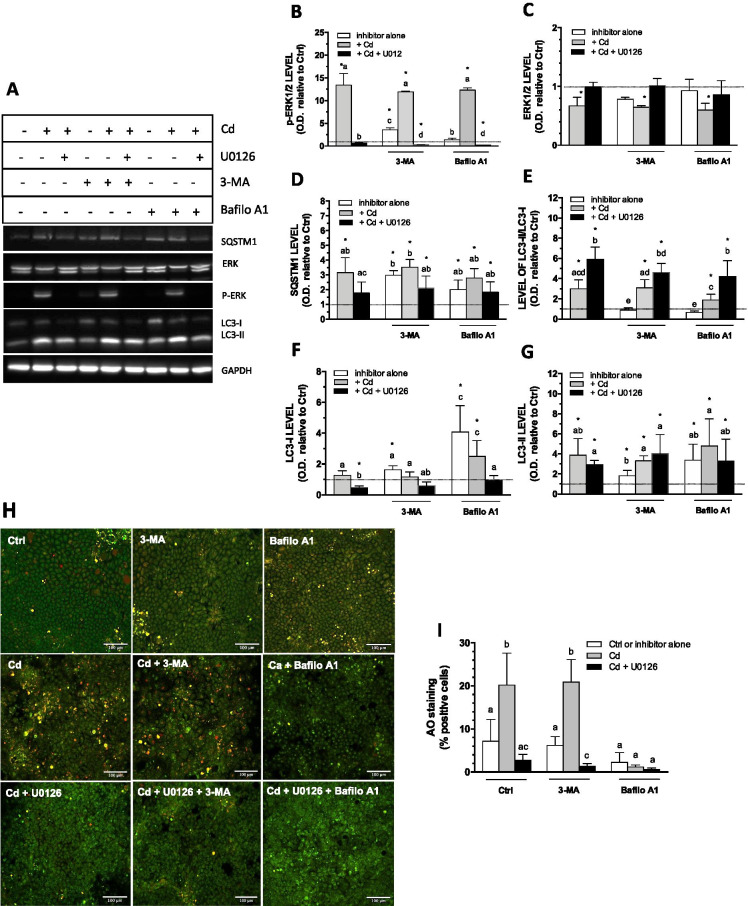
Cd-induced modification in autophagic flux in 21-days-old cells. (**A–G**) Representative Western blot and quantification of ERK1/2 and autophagy related proteins in 21-day-old cells following a 24 h FBS deprivation in the presence of 10 µM Cd, 20 µM U0126, 5 mM 3-MA or 5 nM bafilomycin A1, alone or in combination, relative to that of control cells (FBS-free medium alone for 24 h). (**H**) Visualization of acidic autolysosomes with the dye AO in the presence of inhibitors. (**I**) Quantification of AO-positive cells. Scale bar: 100 µm. Data are means ± SD estimated from 3 independent cell cultures. *Significantly different (*P* ≤ 0.1) compared to levels estimated in control cells. Different letters indicate significant differences for the same protein (*P* ≤ 0.1)

The effect of cadmium on autophagy via ERK1/2 activation was further studied using 3-MA and bafilomycin A1. As expected, Cd increased (up to 12-fold) the levels of p-ERK1/2, which was inhibited by U0126 (Fig. 6A, B). A concomitant increase from 8.5 to 20% in the percentage of AO-positive cells was observed, which was reversed by U0126 (Fig. 6H, I). As for basal constitutive autophagy in 7-days cells, bafilomycin A1 diminished levels of staining, but 3-MA, did not. However, for all tested conditions, the presence of U0126 inhibited the Cd-induced increase in the percentage of AO-positive cells (Fig. 6H, I). Neither 3-MA nor bafilomycin A1 modified the Cd-induced activation of ERK1/2 (Fig. 6B). Surprisingly, moderate increases in the level of p-ERK1/2 were also observed in the presence of 3-MA, but as expected, 3-MA alone, similarly to bafilomycin A1, did not increase cells staining with AO (Fig. 6H, I). As already observed in our previous studies (Gebraël and Jumarie 2015; Lemaire et al. 2020), slight decreases (15%) in total ERK1/2 were observed under conditions of very high levels of activation (100 to 150%) (Fig. 6C). Cadmium, 3-MA, and bafilomycin A1 alone increased SQSTM1 levels, while autophagic inhibitors did not modify the effect of Cd, and the presence of U0126 significantly abolished the Cd-induced increased in SQSTM1 only in the absence of the autophagic inhibitors (Fig. 6D).

**Fig. 7:**
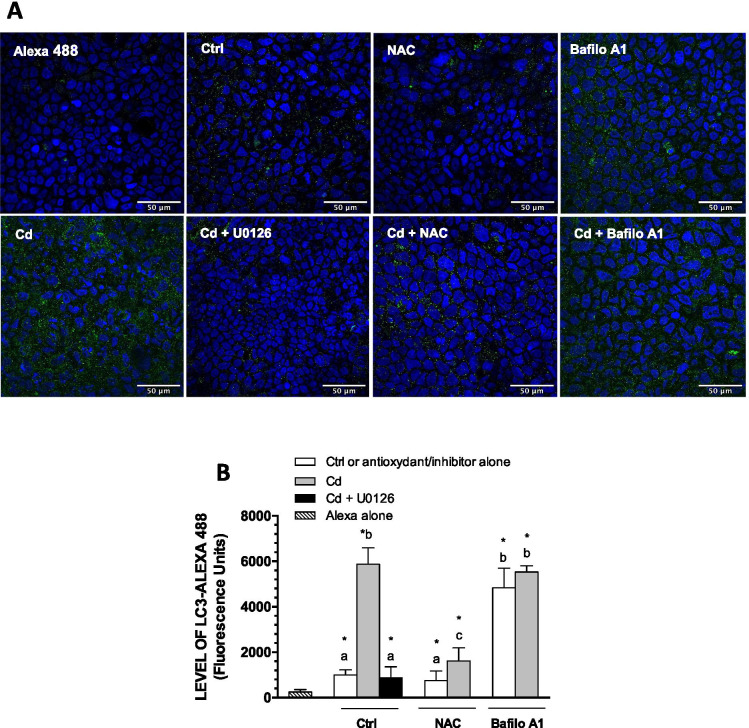
LC3 puncta in 21-day-old cells. (**A**) Cells were grown in the presence of FBS for 21 days, and were then exposed to 10 µM Cd (in the presence or the absence of 20 µM U0126), 1 mM NAC or 3 mM BSO, alone or in combination, for 24 h in FBS-free medium. Cells were then fixed and successively stained with Alexa 488 anti-LC3 antibody (green), DAPI (blue). **B**. Quantification of green puncta. Scale bar: 50 µm. Data are means ± SD estimated from 3 replicates. *Significantly different (*P* ≤ 0.1) compared to levels estimated with Alexa 488 alone (without anti-LC3 antibody). Different letters indicate significant differences (*P* ≤ 0.1)

In accordance with results shown in Fig. 5, Cd increased LC3-II/LC3-I ratio, resulting from a fourfold increase in the level of LC3-II while that of LC3-I remained unchanged (Fig. 6E, F, G). Again, the addition of U0126 lowered LC3-I levels below control values and had a lesser effect on LC3-II, thereby leading to a higher LC3-II/LC3-I ratio. 3-methyladenine or bafilomycin A1 did not modify LC3-II/LC3-I ratios in cells treated with Cd and U0126. Surprisingly, neither 3-MA nor bafilomycin A1 alone modified the LC3-II/LC3-I ratio. However, this might be a consequence of comparable increase in levels of LC3-II and LC3-I in treated cells (cell treatment with bafilomycin A1 alone led to a fourfold increase in the content of each form of LC3) (see Discussion). The hypothetical effect of Cd on autophagy in 21-day-old cell cultures was further investigated by immunofluorescence. As shown in Fig. 7, huge increases in LC3-Alexa puncta were observed following cell treatment with Cd, which was reversed by U0126. In accordance with Western blot analyses, bafilomycin A1 alone also increased LC3-Alexa fluorescence without modifying the effect of Cd. These data hence suggested that Cd modulates the autophagy flux in post-confluent cells through ERK1/2 activation.

### The role of ROS in Cd-induced modulation of autophagy in post-confluent Caco-2 cells

**Fig. 8:**
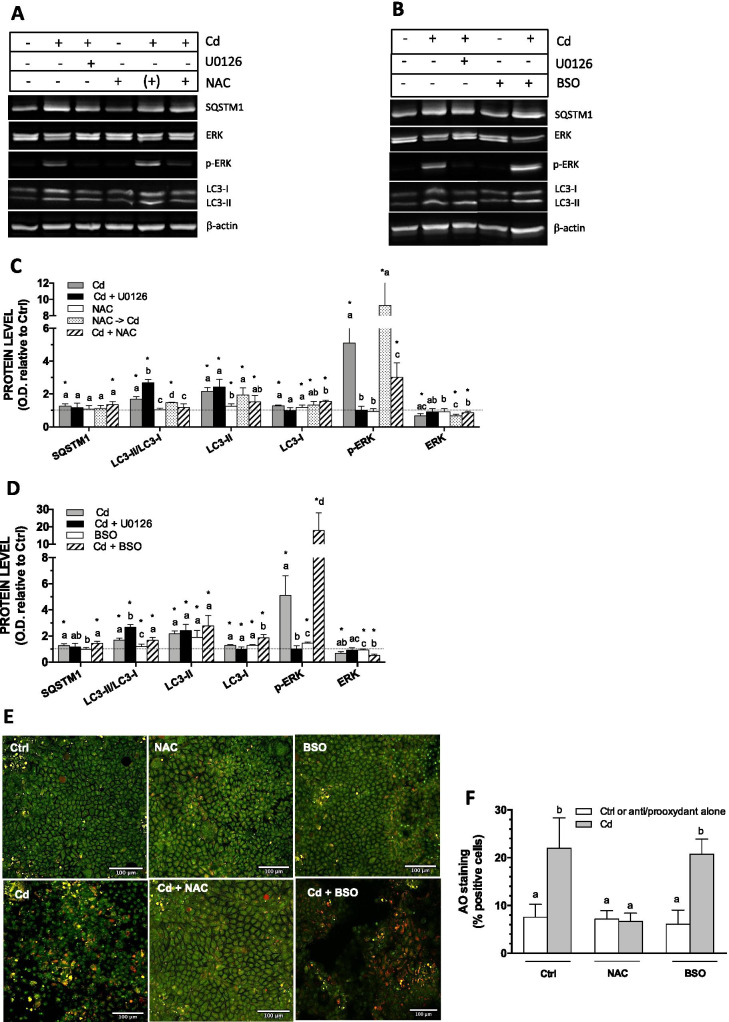
Involvement of ROS in Cd-induced modification in autophagy in 21-day-old cells. Cells were grown in the presence of FBS for 21 days, and were then exposed to 10 µM Cd alone or in combination with 20 µM U0126 or 1 mM NAC or 3 mM BSO for 24 h in FBS-free medium. In some experiments, cells were treated with NAC prior to exposure to Cd (NAC -> Cd). (**A**, **B)** Representative blots. (**C**, **D**) quantification of ERK1/2 and autophagy related proteins relative to that of control cells (FBS-free medium alone for 24 h). (**E**) Visualization of acidic autolysosomes with the dye AO in the presence of Cd, NAC or BSO. (**F**) Quantification of AO-positive cells. Scale bar: 100 µm. Data are means ± SD estimated from 3 to 4 independent cell cultures. *Significantly different (*P* ≤ 0.1) compared to levels estimated in control cells. For the same protein, different letters indicate significant differences (*P* ≤ 0.1)

Cadmium is known to induce oxidative stress and we have previously shown the involvement of ROS in Cd-induced activation of ERK1/2 in Caco-2 cells (Lemaire et al. 2020). Thus, the implication of ROS in Cd-dependent modulation of autophagy was investigated in cells treated with the antioxidant NAC or BSO, which inhibits glutathione synthesis and promotes pro-oxidant conditions. Using DCFH-DA fluorescence to directly measure cellular ROS, we have previously shown that these conditions indeed prevent and favor Cd-induced ROS formation, respectively, in 21-day-old Caco-2 cells (Lemaire et al. 2020). The Cd-induced fivefold increase in p-ERK1/2 was partially inhibited by co-exposure with NAC but not by NAC pretreatment before incubation with Cd (Fig. 8A, C) in accordance with our previous studies showing that pretreatment did not succeed in preventing Cd-induced ROS formation (Lemaire et al. 2020). Interestingly, concomitant exposure of cells with Cd and NAC (but not NAC pretreatment) led to a reduction in LC3-II/LC3-I ratio to control levels (Fig. 8A, C). Also, the addition of NAC during cell exposure to Cd further increased the levels of LC3-I (Fig. 8A, C). Immunofluorescence analyses revealed that NAC importantly lowered the Cd-induced increase in the level of LC3 (Fig. 7). However, NAC had no effect on the modest increase in SQSTM1 in cells exposed to Cd (Fig. 8C). Of note, direct measurement of cellular Cd content by ICP-MS had demonstrated that coincubation with NAC did not result in lower cellular accumulation of Cd (Lemaire et al. 2020). Thus, NAC-mediated alteration on Cd-induced modulation on the levels of autophagic markers is related to its protective effect against Cd-induced ROS formation. Contrary to NAC, co-exposure with BSO enhanced Cd-induced activation of ERK1/2. L-buthionine-sulfoximine alone led to a 75% increase in p-ERK1/2 and a comparable increase in LC3-II levels as that observed in cells exposed to Cd (Fig. 8B, D). N-acetylcysteine completely inhibited increase in the percentage of AO-positive cells, whereas BSO has no effect (Fig. 8E, F). These results show that ROS induction by Cd or BSO alone may activate ERK1/2 and suggest that BSO effect is not sufficient to alter autophagy and that the effect of Cd on autophagy is triggered by a redox signal.

## Discussion

### Autophagy mediated by nutrients or serum deprivation involved ERK activation

Autophagy triggered by nutritional stress and glucose starvation is well documented (Moruno et al. 2012). Generally, for various cell types, a 16 to 24 h exposure to very low level of glucose (0.5 mM) is sufficient to increase LC3-II/LC3-I ratio (Briaud et al. 2003; Roberts et al. 2014). Our study shows that in the Caco-2 cells, a 24 h glucose deprivation is not sufficient despite its impact on the activation of ERK1/2 (Fig. 5). Other investigators also reported longer periods of cells starvation, up to 72 h, for optimal activation of autophagic markers (Nighot et al. 2015). In the present study, a longer time of glucose deprivation (48 h) led to an important and moderate increase in LC3-II and LC3-I, respectively, thereby leading to a substantial increase in LC3-II/LC3-I ratio (Fig. 5) Autophagy leads to the degradation of ubiquitinated proteins that are associated with SQSTM1 which itself interacts with LC3-II for the sequestration of these proteins into the autophagosomes. LC3-II overexpression has been correlated with SQSTM1 degradation (Kambas et al. 2012). However, variation in SQSTM1 is not always related to a modified autophagic flux, and conversely, the level of SQSTM1 may not vary despite strong induction of autophagy (Klionsky et al. 2016). Moreover, prolonged glucose starvation may upregulate SQSTM1 mRNA, which may be the case in our study where a 50% increase in protein level was observed in glucose-deprived cells (48 h) (Fig. 5) (Sahani et al. 2014). Importantly, the transient increase in p-ERK1/2 following a 24 h glucose withdrawal suggest that ERK1/2 activation occurs upstream of the autophagy process. Phospho-ERK1/2 would trigger starvation-induced autophagy but ERK1/2 would not be maintained active during the autophagy process causing a decrease in p-ERK1/2 levels at 48 h. The involvement of p-ERK1/2 is further supported by the absence of AO-positive cells and the significant lower level of LC3-II in cells treated with U0126 during glucose deprivation (Fig. 5). The AMP-activated protein kinase (AMPK) signaling, in response to increased AMP/ATP ratio resulting from energy depletion, may trigger autophagy, notably by inhibiting mTORC1, to restore ATP from cellular components (For review see He et al. 2018). Redox signaling could also be involved in glucose deprivation-mediated autophagy. Indeed, low levels of glucose may induce ROS formation, which leads to the phosphorylation of AMPK, ERK1/2 or JNK with subsequent autophagy induction (Wang et al. 2011, 2009b; Wong et al. 2010). In the present study, the involvement of ROS in the induction of autophagy by glucose starvation was not investigated but our data confirm the involvement of ERK1/2, which may also modulate autophagy induced by amino acid deprivation in the human colonic cell line HT-29 (Ogier-Denis et al. 2000). Our results also provide new insights on the kinetics of ERK1/2 activation and the autophagic response to glucose withdrawal in Caco-2 cells, and support our focus toward autophagy studies in relation to ERK1/2 signaling.

The present study also reveals that serum deprivation may modulate autophagy activity, although moderately. In cell cultures, serum does not represent a source of nutrients but rather provides cells with hormones and growth factors. Because of its critical role in endocrine glands in controlling intracellular production and storage of hormones, autophagy has been studied in endocrine tissues. The signaling pathways modulating the hormonal control of autophagy in specific target tissues is well documented, especially for estrogen-mediated autophagy, with the involvement of AKT or ERK1/2 (Tang et al. 2020; Kurashige et al. 2020). Our results show that, as for nutrient deprivation, serum starvation may also represent a stress that triggers ERK1/2 and autophagy activation. Indeed, higher levels of p-ERK1/2 and LC3-II were observed in 21-day-old FBS-starved cells, whereas LC3-I remained stable for the first 24 h and then decreased, showing net increase in LC3-I conversion to LC3-II (Fig. 3). As observed after glucose starvation, variation in the level of SQSTM1 also correlated with that of LC3-II in serum-starved cells; serum deprivation could act like glucose starvation in increasing mRNA expression of SQSTM1 (Klionsky et al. 2016). In human leukemia K562 cells and in rat hepatoma H4IIE cells, serum deprivation, such as amino acid starvation, was shown to induce autophagy via ERK1/2 activation (Wang et al. 2009b). Interestingly, our data revealed that ERK1/2 activation and increases in SQSTM1 and LC3-II content were synchronized, while p-ERK1/2 levels decreased upon an increase in LC3-II abundance. Thus, as it has been observed with glucose starvation, p-ERK1/2 triggers autophagy following serum deprivation but remains only transiently active.

### ERK1/2 is involved in the constitutive activity of autophagy in preconfluent cell cultures

The growth-related process of intestinal maturation involves sequential activation of MAPKs. Among these, and as it is observed in vivo in the crypt-villus axis, levels of active p-ERK1/2 proteins are high in undifferentiated cells but decrease rapidly as Caco-2 cells begin to differentiate (Aliaga et al. 1999; Gauthier et al. 2001). Similarly to ERK1/2, autophagy is active in undifferentiated cells and it decreases during differentiation (Groulx et al. 2012). Autophagy is a process of self-digestion of organelles and damaged proteins to maintain a positive energy source and the quality of cytosolic components. It has been suggested that restriction of autophagy to the intestinal crypt, a location of high proliferative activity, could represent some protective mechanism that prevents the accumulation of cells damage resulting from active replication (Levine and Klionsky 2004; Mathew et al. 2007). Crosstalk between Ras-Raf-ERK1/2 and PI3K-Akt-mTORC1 pathways is certainly implicated in the regulation of autophagy regulation in intestinal cells (Kaur and Moreau 2021). However, to our knowledge, the role of ERK1/2 as a regulator of non-induced, constitutive autophagy in intestinal cells and in the Caco-2 cells as a function of days of culture has not been much investigated. The present study shows i) AO and GFP-LC3 fluorescence associated with proliferative cells at the periphery of monolayer cellular aggregates, exclusively, in 7-day cultures; ii) the absence of AO fluorescence in 21-day control cells with lower p-ERK1/2 levels; and iii) the abolishment of both AO and GFP-LC3 fluorescence in the presence of U0126 in 7-day cultures, with the concomitant increase in SQSTM1, and the lower levels of both LC3-I and LC3-II (Fig. 1). We have attempted several transfection experiments in 21-day-old cultures unsuccessfully (the integrity of cell monolayers is dependent of the tight junctions), and have not been able to analyze LC3 localization with the GFP-LC3 expression vector. Nevertheless, our results are consistent with: i) the involvement of p-ERK1/2 in basal autophagic activity in preconfluent cell cultures; and ii) decreased autophagic activity in older cultures. The involvement of p-ERK1/2 is further supported by limited autophagic flux in the presence of U0126, thereby rendering these cells insensitive to bafilomycin A1-mediated increase of LC3-II. Based on these results, we thue believe that p-ERK1/2 acts upstream to the autophagic process (Fig. 2H).

Variation in ERK1/2 levels and autophagic activities was shown to correlate as a function of number of days of culture. Our data also show that, similarly to p-ERK1/2, LC3-I decreased with time in culture, whereas LC3-II levels stayed constant, resulting in increased LC3-II/LC3-I ratio in older cultures, which is normally associated with higher autophagic activity. Generally, fewer autolysosomes (resulting from autophagosome and lysosome fusion) correlate with lower levels of LC3-II. However, LC3-II may also locate on non-autophagosome structures that are not subjected to lysosomal degradation (Klionsky et al. 2016; Yoshii and Mizushima 2017). This could explain, at least in part, stable levels of LC3-II observed in 21-day-old cells despite weaker AO fluorescence signal expected to be associated to autolysosome. Variation in LC3 forms similar to that observed in the present study was reported by Tunçer and Banerjee (2017) in Caco-2 cells, while the opposite was observed by Groulx et al. (2012) in the Caco-2/15 clone of the parent cell line. In normal intestinal HIEC cells, LC3-I levels decreased more extensively than that of LC3-II during cell differentiation (Groulx et al. 2012), which also led to higher LC3-II/LC3-I ratio in relation to time in culture. Thus, it appears that net variations in LC3-II/LC3-I ratio should carefully be interpreted, since similar modulation of this ratio may result from different patterns of expression of LC3-II relative to LC3-I. Of note, inhibiting p-ERK1/2 in 7-day-old cells also elicited an increase in LC3-II/LC3-I ratio resulting from a larger drop in LC3-I levels than LC3-II (Fig. 2). The meaning of the respective variations of the LC3 forms deserves to be further investigated. The association of LC3-II with structures other than autophagosomes, and the variation in affinities of the antibody toward each LC3 form with efficiency of detection varying with the level of protein, could complicate the interpretation that can be deduced from analyses of LC3-II/LC3-I ratios.

Higher levels of SQSTM1 were detected in 21-day-old compared to 7-day-old cells (Fig. 1). In the absence of (or lower) autophagic activity, the ubiquitinated proteins as well as SQSTM1 accumulate in the cell and would support higher levels of SQSTM1 with a negative correlation with autophagic activity in the differentiated Caco-2 cells (Klionsky et al. 2016). Significantly higher levels of SQSTM1 were detected in differentiated normal intestinal cells and in the upper third of the colonic crypt of the adult human colon, unlike for the Caco-2/15 cells (Groulx et al. 2012). However, in the present study, variation in the level of SQSTM1 under control conditions was comparable to those reported in the normal tissue (Groulx et al. 2012).

### Cadmium modifies autophagy via ROS-mediated p-ERK1/2 activation in postconfluent cell monolayers

ERK1/2 signaling has been shown to stimulate or to inhibit autophagy in various cells types (Meng et al. 2015; Bastola et al. 2017; Kinsey et al. 2019; Vukelic et al. 2020; Qiao et al. 2021). Different duration and levels of ERK1/2 activation also modulates the fine balance between apoptosis, necrosis and autophagy, and Cd-induced ERK1/2 activation may lead to apoptosis or necrosis (Cheng et al. 2008; Melo-Lima et al. 2015; Xu et al. 2016). In the present study, a 10 µM concentration was chosen for Cd for ERK1/2 activation based on our previous studies (Mantha and Jumarie 2010; Gebraël and Jumarie 2015; Lemaire et al. 2020). This level of exposure was sublethal with a maximal 5–10% cell mortality (the LC_50_ value following a 24-h exposure to Cd has been previously estimated at 140 ± 30 µM; Cardin et al. 2009). In mouse JB6 epidermal cells and in human L02 liver cells, LC3-II augmented as a function of concentration (up to 12 µM) and time (up to 24 h) of exposure to Cd (Son et al. 2011; Pi et al. 2013). In rat pheochromocytoma PC-12 cells, increases in LC3-I conversion to LC3-II was optimal as early as 4 h following a 20 µM exposure to Cd upon which LC3-II decreased rapidly to control levels, clearly demonstrating a transient effect on autophagy (Wang et al. 2013). In these studies, whether Cd was added in culture media containing serum was not specified, and thus limit the comparison to their data. Furthermore, ERK1/2 activation was not investigated. Our results show that maximal conversion of LC3-I to LC3-II as well as optimal level of SQSTM1 occurred 12 to 24 h following exposure to 10 µM Cd (Fig. 4). These effects were sustained, and contrary to what was observed with FBS starvation (and suggested with glucose deprivation), Cd-induced ERK1/2 activation was also maintained throughout the time course. Because transient and sustain activation of ERK1/2 may have completely different effect, it is possible that nutrient- and Cd-induced ERK1/2 activation differently affect autophagy.

A 24-h exposure to 10 µM Cd increased the level p-ERK1/2 with significant increase in the percentage of AO-positive cells, levels of LC3-II and no change in LC3-I (Figs. 5, 6). This may be related to a rapid LC3-I conversion to LC3-II involved in the formation of autophagosome, hence precluding any accumulation of LC3-I (Klionsky et al. 2016; Wang et al. 2013). If we consider that Cd stimulates autophagy, the increase in the level of SQSTM1 remains surprising but could be explained by the fact that Cd activates ERK1/2 in cells with very low level of p-ERK1/2, and that RAF1/Raf-MAP2K/MEK-MAPK/ERK signaling can stimulate SQSTM1 transcription (Klionsky et al. 2016). In fact, inhibiting p-ERK1/2 lowered Cd-induced increase in SQSTM1 although this was not statistically significant (Fig. 6). Alternatively, increases in levels of SQSTM1, LC3-II, and LC3-I could also result from a slower autophagic flux. This possibility is further supported by i) the similar increase in the level of SQSTM1 by Cd, 3-MA and bafilomycin A1; ii) the lack of significant effect of either 3-MA or bafilomycin A1 on Cd-induced increases in autophagic markers, except for LC3-I (Fig. 6). Hence, Cd could inhibit early or late steps of autophagy. The similar increase of LC3-Alexa puncta fluorescence observed with Cd and bafilomycin A1 would rather suggest an inhibitory effect on autolysosome formation (Fig. 7). However, immunofluorescence does not distinguish between LC3 forms, and the effects of bafilomycin A1 and Cd on each of these forms were quite different. Bafilomycin A1 equally increased the levels of LC3-I and LC3-II, hence the resulting constant ratio LC3-II/LC3-I, while Cd increased the level of LC3-II exclusively leading to a threefold higher LC3-II/LC3-I ratio.

Also it is possible that high level of AO staining following Cd exposure would be preferably associated with lysosomes rather than autolysosomes. Hence, Cd would greatly lower lysosomal pH (Fig. 6). This possibility is in accordance with a recent report showing increased lysosomal acidity in the AML12 liver cells following exposure (up to 24 h) to Cd (up to 20 µM) but contrast with others showing lysosomal alkalinization (Wang et al. 2017, 2019; Zou et al. 2020). Some studies have shown that Cd may disrupt autophagic flux by impairing the fusion of autophagosomes with lysosomes leading to increased levels of LC3-I and LC3-II (Wang et al. 2017, 2019; Zhang et al. 2019). Both impaired and enhanced lysosomal degradation capacity have been proposed (Wang et al. 2019; Zou et al. 2020). Moreover, in a single study, Cd has been suggested to induce autophagy but also inhibit its flux (Zou et al. 2020). These conclusions were based on comparisons between the variation in LC3-II and SQSTM1 levels following cell treatment with Cd, bafilomycin A1 or chloroquine, alone or in combination. As we observed, in this study Cd also increased the levels of SQSTM1. Autophagy is a dynamic process whose flux can vary from one cell type to another or according to cell differentiation. It is conceivable that different inducers or inhibitors act on the flux according to their own kinetic, and that a compound modifies the level of autophagic proteins while also affecting the flux in an opposing manner, which obviously complicates the interpretation of results.

The significance of AO fluorescence after exposure to Cd remains to be clarified but the inhibitory effect of U0126 was systematically observed, as with glucose withdrawal (Figs. 5 and 6). These observations confirm the involvement of p-ERK1/2, a conclusion that is further supported by the inhibitory effect of U0126 in the Cd-induced increase in LC3-Alexa fluorescence (Fig. 7). Acridine orange and immunofluorescence analyses also support a role of a redox signal in Cd effects; increases in autolysosome/lysosome staining and LC3-Alexa puncta were counteracted by the antioxidant NAC (Figs. 7 and 8). Cadmium binds to intracellular SH-groups and may favor Fenton reaction leading to oxidative stress and redox imbalance. We have previously shown that a 24-h exposure to 10 µM Cd increases ROS in 21-day-old Caco-2 cells (Lemaire et al. 2020) and that Cd-induced ERK1/2 activation involved redox signaling. The present study confirms that pro-oxidant conditions increase the level of p-ERK1/2, whereas the presence of antioxidants prevent Cd-induced ERK1/2 activation, further showing a positive and a negative correlation, respectively, in terms of LC3-II levels and the LC3-II/LC3-I ratio (Fig. 8). In addition to decreasing LC3-II levels, the presence of NAC also increased LC3-I levels in cells exposed to Cd, showing a net reduction in the conversion of LC3-I to LC3-II. Exposure to BSO alone slightly increased levels of p-ERK1/2, LC3-I and LC3-II, but the redox imbalance would not be sufficient to significantly modify autophagy, as suggested by the lack of AO staining. The effect of Cd on SQSTM1 seems relatively insensitive to the redox state of the cell. Various studies have shown the involvement of ROS-related ERK1/2 signaling in autophagy (Wong et al. 2010; Li et al. 2015; Pan et al. 2015). ROS-mediated autophagy induced by Cd was studied in relation to the energy balance with activation of the glycogen synthase kinaseβ (GSK-3β) in mesangial cells and of AMPK in skin epidermal cells (Son et al. 2011; Wang et al. 2009a). The present study shows that Cd via ROS-induced activation of ERK1/2 may modify autophagy in 21-day-old Caco-2 cells which normally express very low levels of p-ERK1/2 and autophagic activity.

Autophagy is a process that may help cell survival through the degradation of damaged cellular components, although autophagy may also promote cell death through excessive self-digestion. Autophagy in cells exposed to Cd is often considered as protective, but this needs to be confirmed in Caco-2 cells. For example, rapamycin, which activates autophagy through the inhibition of mTORC1, inhibited Cd-induced apoptosis in neuronal and osteoblastic cells (Liu et al. 2016; Xu et al. 2016). On the other hand, following a semi-chronic exposure of 7 days, inhibiting autophagy also alleviated Cd-induced apoptosis and necrosis in immune cells (Gu et al. 2019). Other investigators suggested that Cd-induced autophagy by itself would protect against cell damages resulting from ROS formation, including LDH leakage and DNA fragmentation (Wang et al. 2013, 2015). However, the precise role that ROS play remains to be clarified as increases in cell damages were observed in the presence of NAC, as a result of inhibition of ROS-mediated autophagy, although NAC could also have prevented damages induced by ROS.

## Conclusion

The present study shows i) correlated variation between ERK1/2 and autophagy activities in the human intestinal cells Caco-2 as a function of culture days; and ii) the involvement of ERK1/2 signaling in the regulation of the basal constitutive autophagic flux in proliferative cells, but not in postconfluent cell cultures. In the latter, serum and glucose deprivation triggered autophagy likely via transient phosphorylation of ERK1/2, while Cd activation of ERK1/2 was sustained for at least 36 h with optimal effect on autophagic markers at 24 h. Moreover, Cd effect on autophagy is a ROS-mediated process that is prevented by antioxidant and exacerbated by pro-oxidant conditions. Basal autophagy flux in proliferative cells and Cd-induced increases in autophagic markers in postconfluent cells both involved p-ERK1/2. Constitutive high level of p-ERK1/2 and induced activation of ERK1/2 may have different effects. Whether Cd blocks autophagic flux in older cell cultures remains to be clarified but our data suggest a dual effect. The impact of Cd-induced ERK1/2 activation and the related effect on autophagy on intestinal tissue homeostasis should be studied in the future as i) the predominant role of PI3K/Akt and Raf/MEK/ERK pathways in cell survival vary with differentiation status (Osaki and Gama 2013); ii) ERK1/2 and autophagy activities are finely regulated along the crypt villus axis (Aliaga et al. 1999; Gauthier et al. 2001; Groulx et al. 2012); iii) autophagy plays an important role in the control of the mucosal immune system; impaired autophagy is associated with alteration in gut microbiota and inflammatory bowel disease (Shao et al. 2021; Yang et al. 2018). Our results prompt further studies to better understand the molecular mechanisms responsible for the different and complex effects of Cd on the intestinal cells, which may accumulate and trap high levels of Cd under some nutritional conditions (Eklund et al. 2004; Hiratsuka et al. 1999; Jumarie et al. 1999; Tinkov et al. 2018).

## Data Availability

Not applicable.
